# From competition to protection: mechanisms of mold interactions on bamboo and development of a synergistic natural mold inhibitor

**DOI:** 10.1128/spectrum.03897-25

**Published:** 2026-07-06

**Authors:** Shengnan Zhang, Qichao Bao, Jianliang Ding, Jiawei Zhu, Chungui Du, Pengxu Tao, Xilong Wang

**Affiliations:** 1 Bamboo Industry Institute, Zhejiang A & F University 719309, Hangzhou, People's Republic of China; National Taiwan University, Taipei, Taiwan

**Keywords:** bamboo mold, competitive interaction, cinnamaldehyde, synergistic effect, anti-mold

## Abstract

The mold-induced decay of bamboo is a complex process. Current research on bamboo protection predominantly focuses on single mold species, often overlooking the competitive succession among multiple strains. To address this limitation, we investigated the interspecific competition mechanisms among three common bamboo molds: *Aspergillus niger* (AN), *Trichoderma viride* (TV), and *Penicillium citrinum* (PC). The results indicate that AN dominated spatial colonization due to its robust resource acquisition capabilities and growth advantages. Although TV exhibited rapid growth, it suffered a high rate of cellular inactivation. Conversely, PC demonstrated superior cellular stress tolerance. Based on these competitive dynamics, cinnamaldehyde was selected as the core mold inhibitor from eight natural plant extracts due to its optimal broad-spectrum inhibitory activity. Subsequently, a cinnamaldehyde-citral synergistic formulation was developed, which exhibited pronounced synergistic inhibitory effects, particularly against the highly aggressive AN and TV. In a 28-day anti-mold test, the treated bamboo samples achieved a mold rating of 0, demonstrating a 100% protective efficacy. Microscopic analyses confirmed that this composite inhibitor inactivates mold by disrupting the cellular wall and membrane structures. Furthermore, the retention of this inhibitor system within the bamboo pores is primordially driven by deep physical deposition and robust physical adsorption inside the porous networks, while trace chemical anchoring via potential Schiff base reactions serves as a minor, supplementary mechanism, thereby exhibiting excellent short-to-medium-term protective efficacy under standard conditions. This study provides novel insights and a theoretical foundation for the development of sustainable, high-efficiency natural mold inhibitors for bamboo.

**IMPORTANCE:**

Mold growth on bamboo products causes significant economic losses and poses potential health risks. Current anti-mold treatments often rely on harsh chemicals and overlook the complex, competitive dynamics among different mold species during colonization. This study reveals the interspecific competitive relationships among common bamboo molds, identifying the most aggressive colonizers. Based on these ecological insights, we developed a highly effective, eco-friendly synergistic formulation combining cinnamaldehyde and citral. This compound inhibitor selectively targets dominant molds by disrupting their cellular structures, while achieving reliable short-to-medium-term protection under standard temperature and humidity conditions through the synergy of multi-scale physical entrapment and secondary chemical anchoring. This work provides a rigorous theoretical basis for developing a new generation of targeted, eco-friendly anti-mold technologies for natural lignocellulosic materials.

## INTRODUCTION

As a globally distributed and abundant renewable biomass material (1), bamboo possesses significant advantages, such as being low-carbon and environmentally friendly, high strength, excellent toughness, good biodegradability, and good processability (2–5). Over recent years, the application range of bamboo products in fields such as construction, furniture, and interior decoration has continued to expand (6, 7).

However, bamboo’s structure contains abundant nutrients, such as cellulose, hemicellulose, lignin, as well as starch, sugars, and proteins (8). This makes it highly susceptible to fungal colonization in warm (20°C–35°C) and high-humidity (relative humidity >75%, especially >93% RH) environments (9, 10), particularly by molds such as *Aspergillus* spp., *Penicillium* spp., and *Trichoderma* spp., and specifically *Aspergillus niger* (AN), *Trichoderma viride* (TV), and *Penicillium citrinum* (PC) (11), leading to the phenomenon of bamboo molding. Molding not only degrades the esthetic appearance and mechanical properties of bamboo and its products, causes difficult-to-remove mold stains on the surface, and shortens its service life (12); it may also release allergenic spores hazardous to health, posing potential risks to human health and the environment (13).

Anti-mold strategies for bamboo are primarily divided into two types: endogenous and exogenous. The endogenous approach modifies the inherent properties of bamboo itself. Techniques like thermal (14), chemical (15, 16), and biological (17) modification aim to achieve mold resistance by reducing nutrient availability (e.g., sugars), decreasing wettability, or constructing an antimicrobial framework within the material (18, 19). Conversely, the exogenous approach involves applying external inhibitors, such as mold inhibitors or preservatives, to the bamboo surface (20–22). These inhibitors provide immediate protection by directly killing or inhibiting the growth of mold. However, whether endogenous or exogenous, these conventional methods often exhibit significant limitations (23). Both strategies tend to treat mold as a monolithic threat, focusing primarily on the “bamboo-inhibitor” interface while largely neglecting the complex interactions within the mold ecosystem colonizing the bamboo surface (24, 25). Mold colonization is not an isolated process but rather a dynamic one of competition and succession (26). Therefore, there is an urgent need to customize anti-mold treatment strategies based on the specific characteristics and competitive interactions of the mold species involved.

During the process of bamboo biodeterioration, molds from the genera *Trichoderma*, *Aspergillus*, and *Penicillium* exhibit significant differences in their growth rates, colony morphologies, and metabolic products (12). In natural settings, these molds rarely exist in isolation; instead, they form complex microbial communities that collectively act on the bamboo substrate (27). The competitive interactions among different mold species—such as antagonism (28), resource competition (29), and spatial exclusion (30)—are the pivotal factors that determine which mold species ultimately predominates and leads to the biodeterioration of bamboo (31, 32). Although their individual roles in causing biodeterioration have been studied, the nature of their interactions on a shared substrate and the combined impact of these dynamics on the overall process of bamboo biodeterioration remain poorly understood. This knowledge gap limits a deeper understanding of the bamboo molding. Therefore, an investigation into the interaction mechanisms among these dominant mold species is crucial for elucidating the true process of bamboo biodeterioration and for developing more targeted anti-mold strategies.

The application of a single mold inhibitor often faces challenges when addressing the complex mold communities on bamboo surfaces (6). Different types of inhibitors, based on their unique mechanisms of action—such as cell membrane disruption (33), protein synthesis inhibition (34), or metabolic pathway interference (35)—exhibit varying degrees of efficacy against specific mold species. Consequently, an inhibitor that is highly effective against AN may show minimal activity against TV, and vice versa. This specificity in inhibitory activity makes it difficult for conventional anti-mold methods that rely on a single inhibitor to achieve broad-spectrum and long-lasting protection against mixed-mold infections. Furthermore, when multiple mold inhibitors are used in combination, complex interactions can occur, leading to synergistic (36), additive, or antagonistic effects. A synergistic interaction, which markedly enhances the overall anti-mold efficacy while reducing the total dosage, represents an ideal outcome for inhibitor optimization, potentially lowering costs and environmental impact (37).

To address the gap between single-mold research and actual multi-species infections, this study focused on three common bamboo molds (AN, TV, and PC). Initially, their growth characteristics and interspecific competitive relationships were investigated using dual-culture assays. Subsequently, the Oxford cup method and the fractional inhibitory concentration (FIC) index were employed to screen specific natural inhibitors and develop an optimal compound anti-mold formulation. Furthermore, the micro-ecological competition dynamics and the cellular-level anti-mold mechanisms were elucidated using multi-scale characterizations (including confocal laser scanning microscope [CLSM], scanning electron microscope [SEM], transmission electron microscope [TEM], and Fourier-transform infrared [FTIR]). The practical protective efficacy of the optimal compound system was verified on natural bamboo substrates. Ultimately, this work provides a rigorous scientific basis for developing targeted, eco-friendly bamboo protection technologies based on interspecific competition mechanisms.

## MATERIALS AND METHODS

### Materials

Three-year-old moso bamboo (Phyllostachys pubescens) culms were obtained from Xizhuyuan Bamboo Products Factory in Zhenghe County, Fujian Province. AN, TV, and PC were isolated from naturally occurring bamboo mold by the microbiology research group of Zhejiang A&F University. These three wild-type indicator strains were subsequently authenticated based on colony morphology, conidiophore structures, and physiological growth characteristics according to standard mycological keys, and were maintained as stable laboratory stock cultures for the evaluation. Although molecular confirmation (e.g., ITS rDNA sequencing) was not executed in this specific study, macro-morphological identification and microscopic diagnostic features (such as the distinct colony pigmentations and unmistakable conidiophore architectures of *Aspergillus*, *Trichoderma*, and *Penicillium*) provide sufficient taxonomic rigor and reproducibility for the practical evaluation of anti-mold efficacy on bamboo substrates.

Anhydrous ethanol (AR, 99%) was purchased from Sinopharm Chemical Reagent Co., Ltd. (China). Other chemical reagents, including glutaraldehyde (AR, 50%), cinnamaldehyde (99%), carvacrol (5-isopropyl-2-methylphenol, 99%), citral (99%), tannic acid (99%), eugenol (99%), ionone (99%), nonanal (99%), and paeonol (99%), were obtained from Shanghai Macklin Biochemical Technology Co., Ltd. (China).

### Determination of mold mycelial growth rate

Mycelial discs (5.0 mm diameter) of AN, PC, and TV were aseptically excised from 7-day-old cultures and inoculated at the center of PDA plates. Three replicates were prepared for each mold strain. Cultures were incubated at 28°C and 85% ± 5% relative humidity (RH). The colony diameter was measured every 12 h using the cross method until mycelial coverage reached 90% of the plate surface. The colony radius (R) was calculated by equation 1.


(1) $$R=\frac{D_{1}+D_{2}}{4}$$

where *R* is colony radius (mm), *D*_1_, *D*_2_ are orthogonal colony diameters (mm).

### Dual culture experiment on PDA medium

Mycelial plugs (5 mm diameter) from two distinct mold species were pairwise inoculated on 90-mm PDA plates. Mycelial plugs were placed symmetrically with a 45-mm interval between them. Each experimental group was performed in triplicate. All plates were incubated at 28°C and 85% ± 5% RH. The colony growth in each group was observed at 12, 24, 36, and 48 h post-inoculation.

### Efficacy of mold inhibitors

The inhibitory efficacy of inhibitors against mold was evaluated using the Oxford cup assay. Spore suspensions (80 μL) were uniformly spread on PDA plates. Sterile Oxford cups were placed on the agar surface, and 80 μL of test inhibitors (equal volume) was added to each cup. Plates were incubated at 28°C and 85% ± 5% RH for 48 h. The diameter (mm) of inhibition zones was measured using the cross method (three replicates). The inhibition rate (%) was calculated by equation 2.


(2) $$M=\frac{D_{3}-D_{2}}{D_{3}}\times 100\%$$

where *M* is the inhibition rate (%), *D*_3_, *D*_2_ are the diameters of the inhibition zone and Oxford cup (mm).

### Minimum inhibitory concentration determination

Stock solutions of the respective natural inhibitors were prepared at 512 μg/mL in absolute ethanol. Concentration gradients ranging from 256 to 0.25 μg/mL were established in a 96-well plate using twofold serial dilution, with 100 μL of each diluted solution dispensed per well. Spore suspensions were adjusted to 0.5 McFarland standard (≈1 × 10^6^ CFU/mL) using sterile saline, and 100 μL of this suspension was added to each well containing the anti-mold inhibitor, yielding a final inoculum concentration of 5 × 10^5^ CFU/mL. Control wells included: positive controls (medium with inoculum), negative controls (sterile medium), and solvent controls (medium containing 1% absolute ethanol). All plates were sealed and incubated statically at 28°C and 85% relative humidity for 48 h. The minimum inhibitory concentration (MIC) was defined as the lowest concentration that completely inhibited mold growth, with all experiments performed in triplicate.

### FIC index determination

The combined anti-mold effect was assessed using a checkerboard assay based on the individual MIC values (38). Maximum concentration was set to four times its MIC (4× MIC) and serially diluted twofold to yield concentrations of 2×, 1×, 0.5×, and 0.25× MIC. For 4 × 4 combination testing in 96-well plates, anti-mold inhibitor A was assigned to rows in ascending concentrations. Anti-mold inhibitor B was assigned to columns in ascending concentrations. Each well received 100 μL aliquots of the respective inhibitor concentration, followed by 100 μL of spore suspension. The experiment was performed in triplicate. The FIC index was calculated by equation 3.


(3) $$FIC\;index=\frac{MICA,comb}{MICA,alone}+\frac{MICB,comb}{MICB,alone}$$

The interaction was interpreted as synergistic (FIC ≤ 0.5), additive (0.5 < FIC ≤ 1), indifferent (1 < FIC ≤ 2), or antagonistic (FIC > 2).

### Anti-mold property test

The anti-mold property was evaluated according to the Chinese standard GB/T18261-2013 (China National Standardization Management Committee, 2013b). The mold control effectiveness (MCE) was calculated according to equation 4.


(4) $$MCE=(1-\frac{D_{1}}{D_{0}}\times 100\%)$$

where *D*_1_ is the average infection value (AIV) of the anti-mold treated bamboo, and *D*_0_ is the AIV of the untreated control sample.

### Characterization

The hyphal morphology and structure were observed by an optical super depth-of-field microscope (OSM, VHX-1000C, Japan) and a scanning electron microscope (SEM, Carl Zeiss Jena, Germany). The cell survival was observed by confocal laser scanning microscope (CLSM, Olympus FV3000, Japan). The cell structures were observed by transmission electron microscope (TEM, JEM-2100F, Japan). The chemical structure of the bamboo post-anti-mold treatment was characterized by an FTIR spectrometer (Nicolet Magna-IR 750).

## RESULTS AND DISCUSSION

### Analysis of mycelial extension rates of the three molds

**Fig 1:**
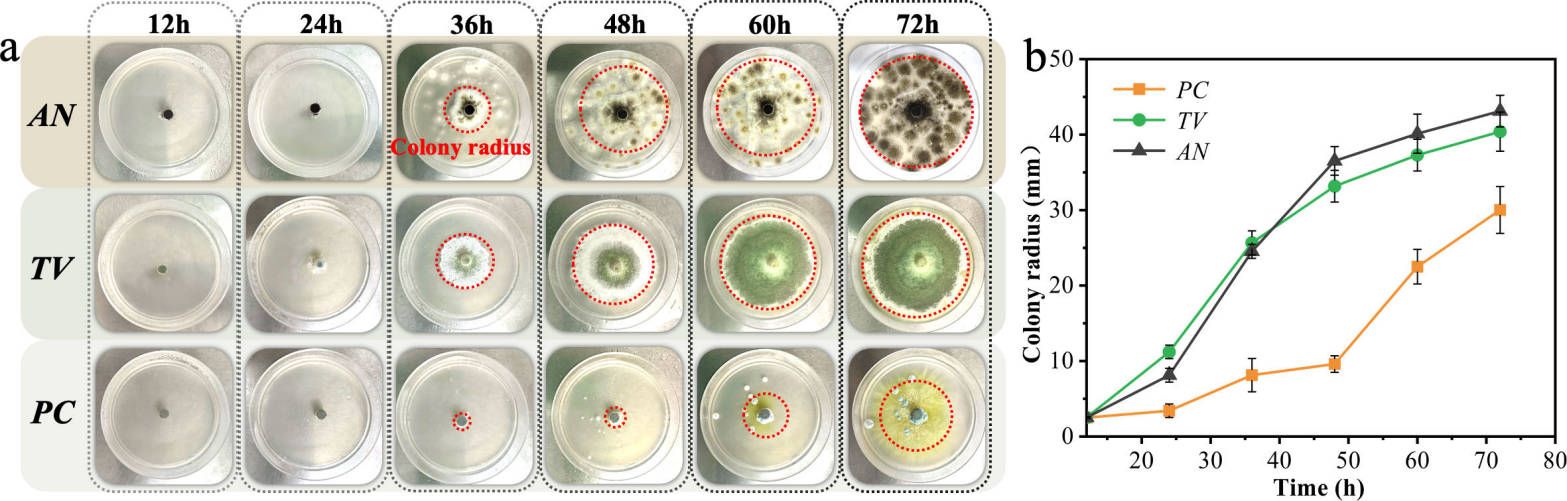
The result of mold growth rates. (**a**) The morphological changes of AN, TV, and PC at different time points. (**b**) Growth rate curve.

The dynamic changes in mycelial extension rates primarily reflect the intrinsic capacity of the molds to utilize survival space and nutritional resources. As shown in Fig. 1, the three molds exhibited markedly different spatial expansion rates when cultured individually. This reflects their unique environmental adaptability and growth characteristics. During the initial 24 h post-inoculation, all species exhibited a distinct lag/adaptation phase, followed by exponential and aggressive radial expansion in AN and TV by 36 h. They rapidly occupied the vast majority of the Petri dish area between 48 and 72 h. In stark contrast, PC demonstrated a relatively sluggish colonization pattern. Its mycelial extension was minimal during the first 48 h, and a larger colony only gradually formed by 72 h (Fig. 1a). The growth rate curves (Fig. 1b) further corroborate these distinct colonization patterns. The growth rates of TV and AN accelerated rapidly during the early stage (12–36 h). They achieved spatial stabilization after 60 h by fully expanding across and covering the available medium. This substantial disparity in individual mycelial extension capabilities suggests potential variations in their underlying growth dynamics. This divergence is postulated to be associated with differing efficiencies in managing endogenous growth factors (e.g., specific metabolic enzymes or signaling molecules). Therefore, the rapid spatial colonization achieved by TV and AN represents a macro-phenotypic advantage, serving as a compelling ecological basis for their independent colonization potentials. Ultimately, these intrinsic differences in growth kinetics reflect their independent colonization potentials. They confer varying initial advantages, setting the stage for the interspecific confrontation dynamics discussed subsequently. The overall spatial colonization capacity decreased in the order of AN, TV, and PC.

### Analysis of competitive advantages among the three molds

**Fig 2:**
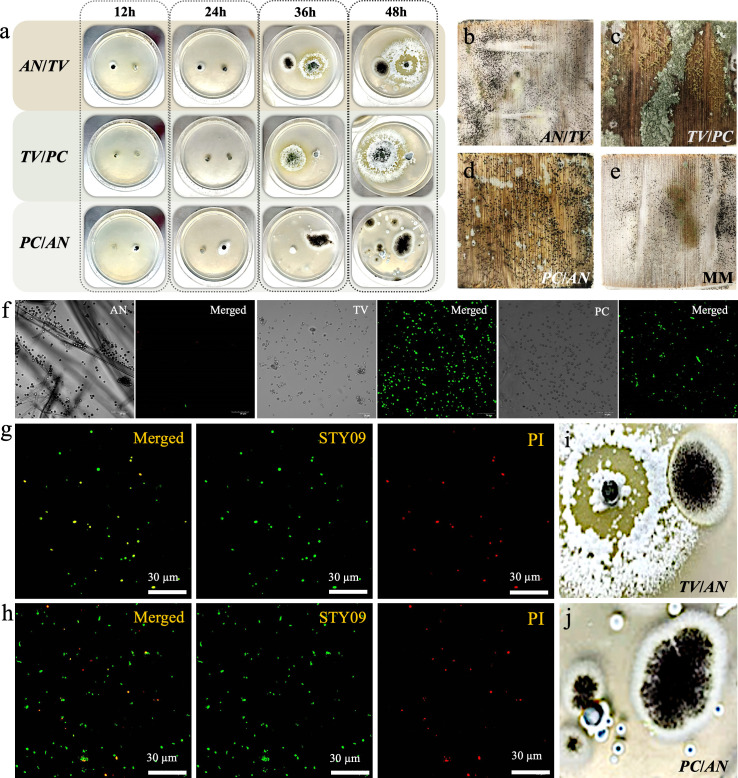
The competition results of three mold species. (**a**) Pairwise confrontation assays of the three molds. (**b–e**) Competitive outcomes of molds on the bamboo substrate. (**b**) AN vs TV; (**c**) TV vs PC; (**d**) PC vs AN; (**e**) MM: mix molds of AN, TV, and PC. (**f**) CLSM images of the three mold species (controls); (**g and h**) CLSM images of TV and PC after competition with AN; (**g**) TV; (**h**) PC; (**i and j**) digital photographs of the TV vs AN and PC vs AN competition.

To investigate the competitive mechanisms among the molds, this study first evaluated their dynamic interactions on culture media using a dual-culture confrontation assay (Fig. 2a). The results indicated that the antagonism between different molds exhibited significant time dependency and species selectivity. Within the initial 12 to 24 h, the colonies expanded independently and gradually approached each other. By 36 h, the hyphae physically intertwined at the interface and began to form an inhibition zone. This suggests potential spatial exclusion or defense responses upon contact, reflecting typical interactive dynamics observed in multi-species ecosystems. By 48 h, the inhibition stabilized and became highly pronounced. Notably, this inhibitory effect demonstrated strong interspecific specificity. Both AN and TV exerted obvious inhibitory effects during competitions, whereas the inhibitory capacity of PC was extremely weak. Overall, AN exhibited the strongest growth inhibition capability and maintained a dominant position in the direct competition for space and nutrients.

This antagonism pattern observed on the culture medium underwent a further divergence in competitive dominance during colonization on natural bamboo substrates (Fig. 2b through e). When the three molds were co-inoculated onto the bamboo surface, AN rapidly formed a dense and prominent colony distribution due to its robust invasive capacity. It occupied a clear ecological advantage in competition with both TV and PC. The colonization ability of TV ranked second, whereas PC was at a distinct disadvantage. This indicates that in a real, lignocellulose-rich environment, AN exhibits a distinct capacity to rapidly establish a population advantage on the bamboo substrate, presumably facilitated by adaptive resource utilization or specialized enzymatic deployment.

To elucidate the microscopic mechanisms underlying this macroscopic competition at the cellular level, cell viability at the competition interface was quantitatively analyzed. This was achieved using CLSM coupled with SYTO-9 and propidium iodide (PI) dual-fluorescent probes. SYTO-9 penetrates intact cell membranes to emit green fluorescence, representing live cells. In contrast, PI only enters cells with damaged membranes to emit red fluorescence, representing metabolically inactivated cells. The experimental results showed that AN could not be effectively stained (Fig. 2f). This is presumably attributable to its unique cell wall structure (e.g., greater thickness and high polysaccharide content) and lower cell membrane permeability. These structural barriers effectively block the entry of dye molecules and restrict their binding to nucleic acids. This observation underscores the strong physical defense capabilities of AN.

Quantifying the cellular states of TV and PC after interacting with AN (Fig. 2g through j) revealed their differentiated ecological adaptation strategies. Following the interaction with AN, up to 31.88% of TV cells exhibited red fluorescence, indicating metabolic inactivation. This demonstrates that although TV grows rapidly at the macroscopic level, it suffers a higher cell inactivation rate during microscopic interspecific confrontations. In contrast, the green fluorescence of PC cells was widely distributed with high intensity. Its metabolic inactivation rate was only 25.82%. This proves that despite its slow mycelial extension and disadvantage in macroscopic competition, PC exhibits a significant “stress-tolerant” trait. It likely develops a certain tolerance to the antagonistic substances secreted by AN through its metabolic regulation, enzymatic system responses, or specific cell membrane structure. Consequently, it successfully maintains a higher cell survival rate.

In summary, the bamboo molding process is co-driven by molds with diverse ecological adaptation strategies. Therefore, an efficient anti-mold strategy for bamboo should specifically target and effectively inhibit the dominant species (AN and TV) that exhibit persistent, rapid growth and strong invasive capabilities.

### Screening and inhibitory performance analysis of natural mold inhibitors

**Fig 3:**
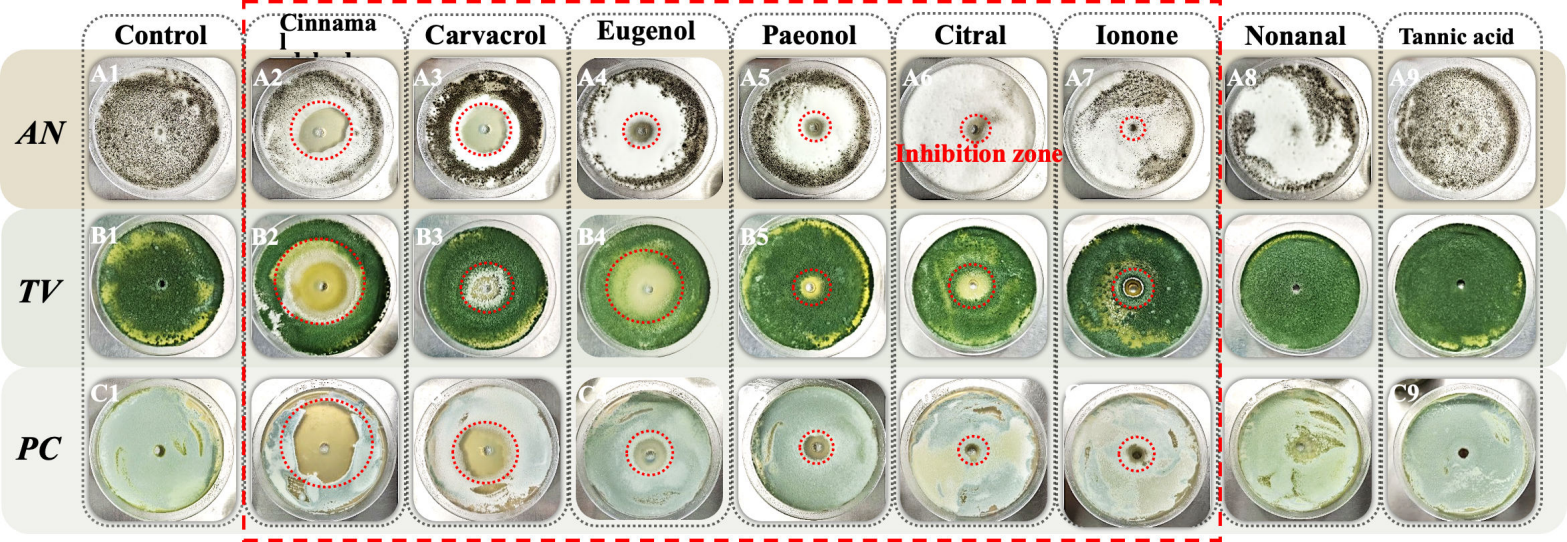
Inhibitory effects of eight natural mold inhibitors on the growth of bamboo molds.

Based on the analysis of mold competition and colonization dominance, an efficient anti-mold system for bamboo must possess broad-spectrum inhibitory capabilities. In particular, it must effectively inhibit the highly invasive AN and TV. This study first conducted a preliminary screening of eight natural plant extracts using the Oxford cup method (Fig. 3 and Table 1). The results showed that nonanal and tannic acid exhibited no obvious inhibitory effects against the three molds. Consequently, they were excluded from subsequent formulation studies. The remaining six plant-derived active monomers all demonstrated significant inhibitory activities. Among them, cinnamaldehyde exhibited the optimal broad-spectrum inhibitory potential. Its inhibition rates against PC, TV, and AN reached as high as 83.76%, 82.65%, and 72.80%, respectively. Carvacrol showed a prominent inhibitory effect against AN (76.76% inhibition rate), whereas citral displayed a highly targeted inhibitory capacity against TV (69.76% inhibition rate). This specific differentiation in inhibitory efficacy is likely related to the varying affinities and destructive mechanisms of different active molecules (e.g., aldehyde groups and phenolic hydroxyl groups) toward distinct mold cell walls and membrane structures (39, 40).

**TABLE 1:** Effect of eight mold inhibitors on the inhibition rate of mold on bamboo

Inhibitor	Inhibition rates (%)		
	AN	TV	PC
Cinnamaldehyde	72.80	82.65	83.76
Citral	14.28	69.76	36.13
Carvacrol	76.76	68.08	72.54
Eugenol	48.76	64.32	65.65
Paeonol	55.13	51.28	56.94
Ionone	0	49.12	15.04
Nonanal	0	0	0
Tannic acid	0	0	0

To quantify the inhibitory efficacy of these natural inhibitors, their MICs were determined using the broth microdilution method (Table 2). This quantitative analysis further confirmed the aforementioned interspecific specificity. Against the dominant colonizer AN, eugenol and carvacrol exhibited exceptionally strong inhibitory activities (both with an MIC of 0.15 mg/mL). Against the rapidly growing TV, citral demonstrated high sensitivity (MIC as low as 0.10 mg/mL). However, when addressing actual mixed-mold infections on bamboo, a single mold inhibitor often struggles to achieve comprehensive inhibition of the entire complex mold community. Comprehensively evaluating all parameters, cinnamaldehyde maintained high and balanced inhibitory activities against all three molds (MICs ranging from 0.20 to 0.25 mg/mL). Furthermore, as an unsaturated aromatic aldehyde, previous studies indicate that its molecular structure endows it with robust lipophilicity and cell barrier-penetrating capabilities (41, 42). Therefore, cinnamaldehyde was ultimately selected as the core foundational inhibitor for constructing the composite anti-mold system for bamboo.

**TABLE 2:** MIC of the mold inhibitor against three mold species

Inhibitor	MIC (mg/mL)		
	AN	TV	PC
Cinnamaldehyde	0.25	0.20	0.24
Citral	0.40	0.10	0.30
Carvacrol	0.15	0.40	0.20
Eugenol	0.15	0.80	0.40
Paeonol	0.40	0.80	0.40
Ionone	0.60	3.20	1.60

### Synergistic effects and formulation optimization of the compound anti-mold system

In the screening of single anti-mold inhibitors, cinnamaldehyde was identified as the most effective inhibitor. We used the checkerboard assay and the FIC index to systematically evaluate the combined inhibitory efficacy of cinnamaldehyde with other natural inhibitors (Table 3). The results showed significant interaction variations among different inhibitor combinations against the three bamboo molds. Among all tested combinations, the mixture of cinnamaldehyde and citral demonstrated the optimal broad-spectrum combined inhibitory potential. This combination exhibited pronounced synergistic effects against the highly invasive and destructive dominant colonizers, AN and TV. The corresponding FIC values were 0.50 and 0.38, respectively. Furthermore, their combined inhibitory concentrations were markedly lower than those of individual applications. In contrast, combinations of cinnamaldehyde with other compounds (such as eugenol, carvacrol, or paeonol) showed indifferent or even antagonistic effects (FIC > 2.0) against specific molds, especially TV. Based on the aforementioned analysis of mold competition dynamics, AN and TV are the primary factors accelerating bamboo degradation. Therefore, the cinnamaldehyde-citral combination was determined as the optimal anti-mold formulation. This compound system can produce strong synergistic inhibitory effects against these two dominant molds.

**TABLE 3:** Combined anti-mold activity of the mold inhibitors against three mold species

	Inhibitor		Alone MIC (mg/mL）		Combined MIC (mg/mL）		FIC	Result
Mold	A	B	A	B	A	B		
AN	Cinnamaldehyde	Citral	0.25	0.40	0.06	0.10	0.50	Synergy
AN	Cinnamaldehyde	Eugenol	0.25	0.15	0.09	0.06	0.74	Additive
AN	Cinnamaldehyde	Carvacrol	0.25	0.15	0.15	0.09	1.20	Indifference
AN	Cinnamaldehyde	Ionone	0.25	0.60	0.19	0.45	1.51	Indifference
AN	Cinnamaldehyde	Paeonol	0.25	0.40	0.15	0.24	1.21	Indifference
TV	Cinnamaldehyde	Citral	0.20	0.10	0.04	0.02	0.38	Synergy
TV	Cinnamaldehyde	Eugenol	0.20	0.80	0.22	0.88	2.21	Antagonism
TV	Cinnamaldehyde	Carvacrol	0.20	0.40	0.30	0.60	3.00	Antagonism
TV	Cinnamaldehyde	Ionone	0.20	3.20	0.07	1.09	0.68	Additive
TV	Cinnamaldehyde	Paeonol	0.20	0.80	0.24	0.94	2.35	Antagonism
PC	Cinnamaldehyde	Citral	0.24	0.30	0.09	0.11	0.74	Additive
PC	Cinnamaldehyde	Eugenol	0.24	0.40	0.06	0.09	0.47	Synergy
PC	Cinnamaldehyde	Carvacrol	0.24	0.20	0.15	0.12	1.22	Indifference
PC	Cinnamaldehyde	Ionone	0.24	1.60	0.14	0.91	1.14	Indifference
PC	Cinnamaldehyde	Paeonol	0.24	0.40	0.29	0.49	2.45	Antagonism

### Anti-mold performance of the compound inhibitor on bamboo

**Fig 4:**
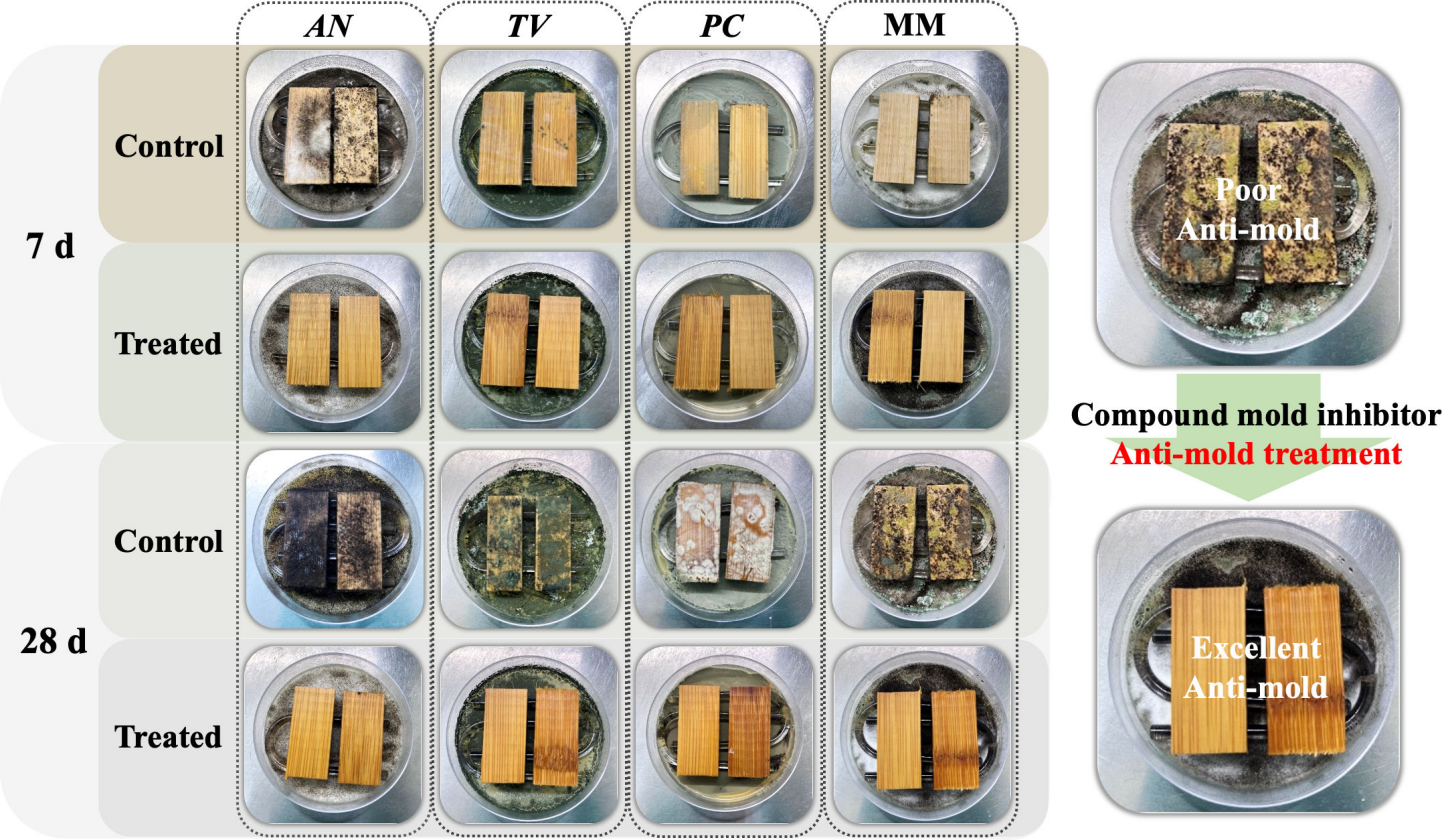
Results of the 28-day anti-mold test.

To further verify the actual effect of the selected compound anti-mold system, bamboo was treated using a room-temperature (~25°C) pressure impregnation process. The process parameters included a compound concentration of 30.0 mg/mL (cinnamaldehyde to citral mass ratio of 2:1), a pressure of 0.3 MPa, and a duration of 90 min. The 28-day anti-mold test was then conducted according to the national standard (GB/T 18261-2013) (Fig. 4). The results showed that the untreated control bamboo rapidly became a substrate for mold colonization under the test conditions. By day 7, obvious signs of mycelial growth appeared on the control surfaces. By day 28, their surfaces were completely covered by PC, TV, AN, and the mixed molds. The infection levels all reached the maximum rating of 4. This phenomenon further confirmed the high susceptibility of unprotected bamboo to these dominant molds. In contrast, the bamboo treated with the compound inhibitor exhibited significant and stable resistance to infection. During both the 7-day initial evaluation and the 28-day standard anti-mold test, no signs of mold spore germination or mycelial extension were observed on the treated samples. Against the three individual molds and the mixed mold community, this compound system consistently achieved a mold rating of 0. The protection efficacy reached 100%. This validation result indicates that the compound inhibitor effectively blocked the colonization behavior of molds on the bamboo surface. It provides a reliable protective basis for the practical application of bamboo in mold-susceptible environments.

### Cellular-scale analysis of mold competition and anti-mold mechanisms

**Fig 5:**
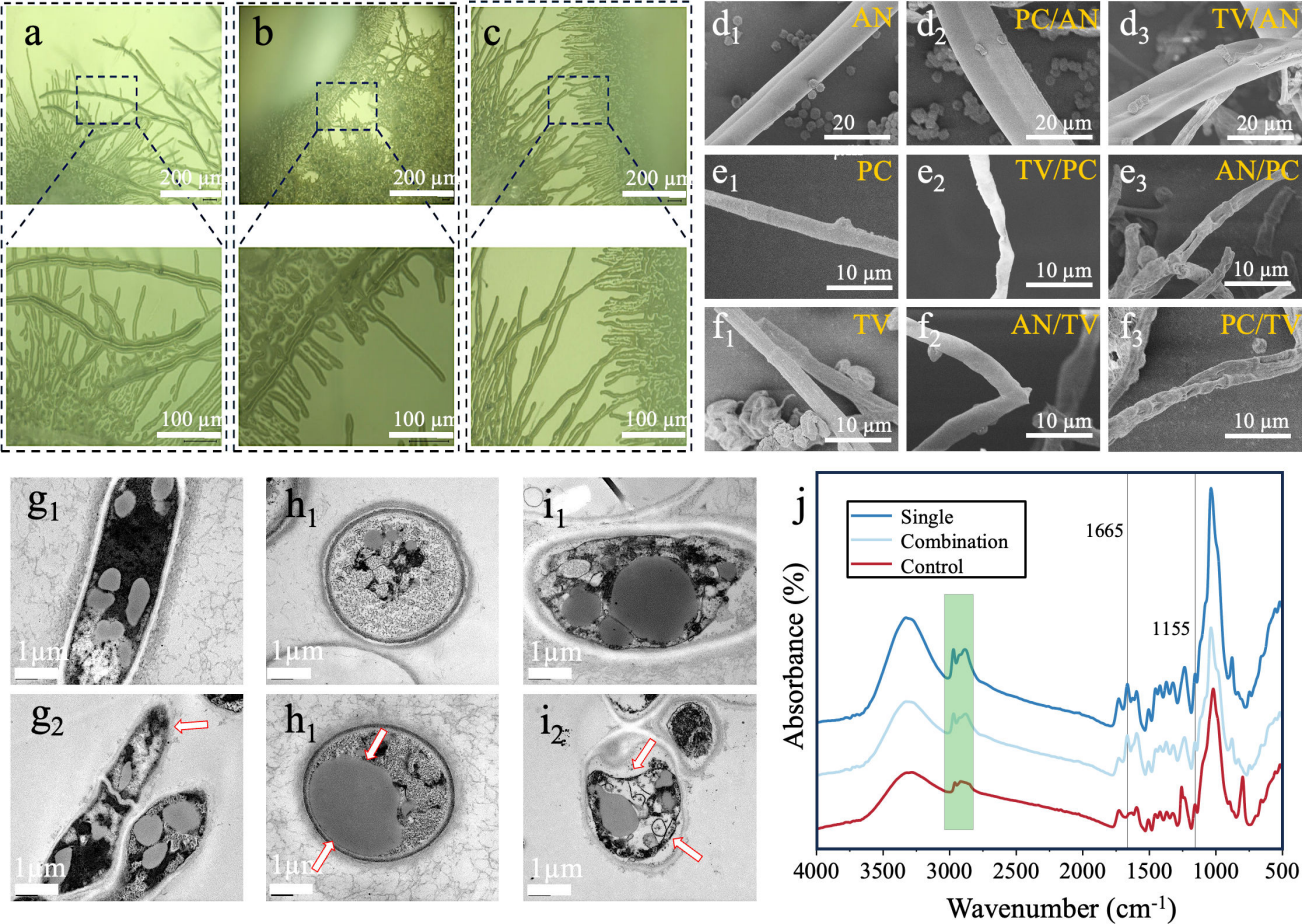
Microscopic characterization of mold competition and inhibition. (**a–c**) Microscope images of mold competition. (**a**) PC vs TV; (**b**) PC vs AN; (**c**) TV vs AN. (**d–f**) Scanning electron microscopy micrographs of mold competition. (**g–i**) TEM micrographs of treated bamboo. (**g**) PC: control (g_1_) and MIC-treated group (g_2_). (**h**) TV: control (h_1_) and MIC-treated group (h_2_). (**i**) AN: control group (i_1_) and MIC-treated group (i_2_). (**j**) FTIR spectra of the bamboo treated with the mold inhibitor. Single: samples impregnated with cinnamaldehyde alone; combination: samples synergistically treated with cinnamaldehyde and citral; control: untreated samples.

This study used ultra-depth-of-field microscopy and SEM to characterize the molds (Fig. 5a through f). These results further revealed the complex antagonistic strategies among the molds at the microscopic scale. The observations showed that different dominant molds adopted distinct spatial and resource-predatory mechanisms. TV primarily exhibited significant mycoparasitism features. Its hyphae coiled around the hyphae of AN (Fig. 5c). Furthermore, they directly invaded the interior of PC hyphae, inducing localized swelling and branching (Fig. 5a). Ultimately, this caused severe shrinkage, breakage, and structural collapse of the competitor’s hyphae (Fig. 5d and e). In contrast, AN relied more on physical spatial suppression. Its hyphae grew rapidly at a high density. This formed a large-area coverage and envelopment over PC, restricting its further expansion (Fig. 5b). In these competitive interactions, the morphology of PC suffered the most severe damage (Fig. 5e). Both AN and TV displayed active attacking and inhibitory behaviors. This confirms the previous macroscopic competition conclusions regarding the dominant colonizers from a microscopic morphological perspective.

To elucidate the lethal mechanisms of the compound inhibitor, treated mold cells were observed using TEM. This observation was conducted at the minimum inhibitory concentration (Fig. 5g through i). The control molds all presented intact cellular morphologies, continuous cell wall-membrane complexes, and evenly distributed intracellular macromolecules. However, under the action of the compound inhibitor, the subcellular structures of all three molds suffered severe and irreversible damage. Specifically, the cell wall of PC became markedly thinner and translucent. Its wall-membrane complex exhibited pathological loosening (Fig. 5g2). TV showed localized cell wall dissolution. This caused a massive leakage of cytoplasmic contents through the rupture. Additionally, internal vacuolization appeared, suggesting the disintegration of key organelles such as mitochondria (Fig. 5h2). AN exhibited cell wall dissolution and membrane reorganization barriers. Its hyphal morphology became distorted and collapsed (Fig. 5i2). This multidimensional structural damage indicates that the cinnamaldehyde-citral compound system can destroy the physical barriers of fungi. It triggers intracellular osmotic pressure imbalance and functional network collapse, ultimately leading to mold inactivation.

In addition to the direct physical destruction of mold cells, the protective efficacy of this compound inhibitor under standard laboratory conditions is associated with the synergistic effect of deep physical entrapment and potential interfacial anchoring. FTIR spectroscopy analysis (Fig. 5j) showed a new characteristic absorption peak at 1,665 cm^−1^ in the treated bamboo. This peak corresponds to the characteristic absorption region of conjugated C=N bonds (1,550–1,650 cm^−1^). This newly emerged peak suggests a potential supplementary Schiff base interaction between the active aldehyde groups of cinnamaldehyde and the trace-free amino groups within the bamboo matrix. Nevertheless, given the inherently low content of nitrogenous compounds or free amino groups in natural lignocellulosic substrates, this chemical cross-linking is deduced to act as a minor, secondary fixation pathway. The sustained resistance of the composite inhibitor within the bamboo pores is primordially driven by deep physical deposition, capillary entrapment, and robust physical adsorption inside the complex porous networks of bamboo generated during the pressure impregnation process. Nevertheless, due to the volatile nature of cinnamaldehyde and citral essential oils, further investigations evaluating the long-term anti-leaching stability and anti-volatilization performance of the treated bamboo under open environments are critically required and represent a paramount direction for future research.

### Conclusion

In this study, macroscopic phenotypic analyses (dual-culture assays, the Oxford cup method, MIC, and FIC) and microscopic/molecular characterizations (ultra-depth-of-field microscopy, CLSM, SEM, TEM, and FTIR) were employed to systematically investigate the interaction mechanisms among three common bamboo molds (PC, TV, and AN) during competition. Furthermore, the protective efficacy and underlying mechanisms of a synergistic natural mold inhibitor were evaluated. The results reveal significant mechanistic differences among the molds during competitive colonization. Specifically, AN demonstrated a robust and persistent growth advantage, effectively inhibiting TV and PC through rapid resource appropriation. While TV grew rapidly, it suffered a higher rate of cellular inactivation during contact-mediated competition. In contrast, PC displayed greater cellular stress tolerance despite its slow mycelial extension. Given their strong infection capabilities on the bamboo substrate, AN and TV were identified as the primary target strains for anti-mold treatments. Guided by these competitive mechanisms, a cinnamaldehyde-citral compound was determined as the optimal anti-mold formulation. This compound exhibited pronounced synergistic inhibitory effects against AN and TV, achieving 100% protective efficacy on bamboo after a 28-day anti-mold test. At the microscopic level, the compound efficiently disrupted the mold cell wall-membrane complexes. Although FTIR analysis implied an auxiliary chemical anchoring via potential Schiff base reactions, the dynamic leaching rates, anti-weathering stability, and desorption kinetics of the physically adsorbed inhibitors under extreme natural environments remain to be comprehensively evaluated. Nevertheless, this study provides a rigorous theoretical basis and innovative insights for developing eco-friendly bamboo mold inhibitors based on synergistic effects.

## Data Availability

All data generated or analyzed during this study are included in this published article. Any remaining raw data that support the findings of this study are available from the corresponding author upon reasonable request.
